# Dysregulation of placental ABC transporters in a murine model of malaria-induced preterm labor

**DOI:** 10.1038/s41598-019-47865-3

**Published:** 2019-08-07

**Authors:** K. N. Fontes, M. W. Reginatto, N. L. Silva, C. B. V. Andrade, F. F. Bloise, V. R. S. Monteiro, J. L. Silva-Filho, G. E. Imperio, P. M. Pimentel-Coelho, A. A. S. Pinheiro, S. G. Matthews, E. Bloise, T. M. Ortiga-Carvalho

**Affiliations:** 1 Laboratory of Translational Endocrinology, Institute of Biophysics Carlos Chagas Filho, Federal University of Rio de Janeiro, Rio de Janeiro, Brazil; 2 Laboratory of Immunology and Biochemistry of Parasitic Diseases, Institute of Biophysics Carlos Chagas Filho, Federal University of Rio de Janeiro, Rio de Janeiro, Brazil; 3 Laboratory of Cellular and Molecular Neurobiology, Institute of Biophysics Carlos Chagas Filho, Federal University of Rio de Janeiro, Rio de Janeiro, Brazil; 4 Department of Physiology, Faculty of Medicine, University of Toronto, Toronto, Canada; 5 Department of Obstetrics & Gynaecology, Faculty of Medicine, University of Toronto, Toronto, Canada; 6 Department of Medicine, Faculty of Medicine, University of Toronto, Toronto, Canada; 7 Lunenfeld-Tanenbaum Research Institute, Mount Sinai Hospital, Toronto, Canada; 8 Department of Morphology, Federal University of Minas Gerais, Belo Horizonte, Brazil; 9 Laboratory of Tropical Diseases, Department of Genetics, Evolution, Microbiology and Immunology, Institute of Biology, State University of Campinas, Campinas, Brazil

**Keywords:** Disease model, Malaria

## Abstract

Malaria in Pregnancy (MiP) is characterized by placental accumulation of *Plasmodium*-infected erythrocytes, intrauterine growth restriction (IUGR) and preterm delivery (PTD). Placental ATP-binding cassette (ABC) transporters mediate the efflux of nutrients, cytokines and xenobiotics. The expression and activity of these transporters are highly responsive to infection. We hypothesized that MiP would perturb the expression of placental ABC transporters, promoting PTD. Peripheral blood, spleens, livers and placentas of pregnant mice, infected with *Plasmodium berghei ANKA* on gestational day (GD) 13.5, were collected and analyzed on GD18.5. The primary consequences of human MiP, including IUGR, PTD (20%) and placental inflammation, were recapitulated in our mouse model. Electron microscopy revealed attenuated presence of labyrinthine microvilli and dilated spongiotrophoblasts -granular endoplasmic reticulum cisternae. Additionally, a decrease in placental *Abca1 (*ABCA1), *Abcb1b (*P-glycoprotein), *Abcb9* and *Abcg2 (*BCRP) expression was observed in MiP mice. In conclusion, MiP associated with PTD impairs placental ABC transporters’ expression, potentially modulating placental nutrient, environmental toxin and xenobiotic biodistribution within the fetal compartment, and may, at some degree, be involved with pregnancy outcome in MiP.

## Introduction

Preterm delivery (PTD) affects 8 to 12% of all pregnancies worldwide, with this number raising up to, approximately, 19% in some low and middle income countries^1,2^. The most common risk factors for spontaneous PTD include multiple gestations, use of toxic substances and intrauterine infection. The latter, accounts for approximately 40% of all cases of PTD globally^3,4^.

Intrauterine infection, caused by various infective agents (bacteria, viruses and protozoa), promotes inflammation of the maternal-fetal unit, which occurs through the recognition of microbial antigens by the pattern recognition receptors (PRRs), and subsequent release of pro-inflammatory mediators capable of inducing labor^5^. Among these, an important infective disease is Malaria, a life-threatening disease that in 2016 affected 216 million people and caused approximately, 445.000 deaths globally^6^.

Malaria in Pregnancy (MiP) is highly associated with an increased risk of intrauterine growth restriction (IUGR), PTD, low birth weight, infant death and adverse postnatal cognitive and neurosensory development^7^. In fact, it is estimated that 125 million pregnant women reside in areas at risk of contracting MiP^8^. The average maternal mortality rate induced by MiP (which can be associated with severe anemia, hypoglycemia, acute respiratory distress syndrome, renal failure and cerebral malaria) is 39%. Importantly, up to 70% of IUGR and 36% of preterm delivery cases in malaria-endemic areas are related to *Plasmodium sp*. infection^8^.

In humans, the pathogenesis of placental malaria includes accumulation of *Plasmodium falciparum*–infected erythrocytes into the intervillous space of the placenta. This culminates in the infiltration of placental immune cells, deposition of malarial pigment (hemozoin) in the placenta, thickening of the placental basement membrane, perivillous fibrinoid deposits and abnormal syncytial knotting. Interestingly, placental infection with *Plasmodium vivax* was shown to be associated with a much milder phenotype^7,9^. Furthermore, the pro-inflammatory placental microenvironment elicited by malaria sequestration is capable of compromising active transporter systems. As such, impaired expression and activity of amino acid transporters have been reported in human and experimental placental malaria and were correlated with increased rates of IUGR and low birth weight, which are both considered to be important consequences of MiP^10,11^.

Previous work also showed that BALB/c mice infection with *Plasmodium berghei ANKA* (from gestational day (GD) 13.5–19.5) was capable of impairing the placental expression of other active transporters belonging to the ATP-binding cassette (ABC) transporter family^12^. However, the pattern of placental ABC transporter expression, in a mouse model where PTD and low birth weight are simultaneously recapitulated, have not been investigated.

Functionally, ABC transporters are responsible for the movement of numerous physiological and pharmacological substrates across developing biological barriers (i.e. the placenta and fetal blood brain barrier). Among the ABC transporters, the best described are the ABC lipid transporter, ABCA1, and the ABC drug transporters, P‐glycoprotein (P‐gp, encoded by *ABCB1* in humans and *Abcb1a/Abcb1b* in rodents), breast cancer resistance protein (BCRP, *ABCG2*) and the multidrug resistance‐associated proteins (MRPs‐2 and 5, *ABCC2* and *ABCC5*). Physiological substrates of ABC transporters include: cholesterol, folic acid, steroid hormones and inflammatory mediators; whereas bisphenol A, many herbicides and pesticides, antibiotics, antiretrovirals and antidepressants represent nonphysiological substrates^13,14^. Thus, it is likely that altered placental ABC transporter expression caused by MiP could disturb the fetal biodisposition of these substrates with potentially significant effects on clinical outcomes. Previous studies from our group have demonstrated that chorioamnionitis, as well as bacterial and viral challenges, alter the expression and activity of P-gp and BCRP in the human placenta, resulting in fetuses being exposed to unbalanced levels of nutrients, xenobiotics and environmental toxins present in the maternal circulation^15–18^.

Given the importance of ABC transporters in fetal nutrition and protection, and due to the fact that experimental malaria alters the placental expression of key ABC transporters, we hypothesized that the dysregulation of ABC transporters is, at least partially, involved in the pathogenesis of MiP-induced PTD and associated low birth weight. In the present study, therefore, using an experimental cerebral malaria strategy^19^ induced by *Plasmodium berghei ANKA* infection in C57BL/6 mice, a new murine model of MiP-induced PTD was developed, to probe whether the expression of key placental ABC transporters is associated with the pathological and clinical features of human malaria during pregnancy^20^.

## Results

### Pregnancy-associated malaria induces preterm delivery and intra uterine growth restriction

Pilot studies were conducted to determine the appropriate infected-erythrocyte regimen necessary for eliciting PTD in 10–20% of the animals, on GD17.5. This stage of gestation was selected based on evidence that murine term birth occurs between GD18 and 22, with an average gestational period of 19.25 days for C57BL/6 mice^21^. Importantly, C57BL/6 birth occurs at GD18.5 in our animal facilities, which is compatible with the average birth age observed for term deliveries in the C57BL/6 lineage elsewhere^21^. In addition, C57BL/6 concepti on GD17.5, exhibit fetal development markers consistent with infants born prematurely^22^. Thus we considered that all C57BL/6 births occurring in our facilities prior to 18.5 were to be classified as PTD.

Pregnant mice were acutely exposed to 1 × 10^5^, 5 × 10^5^ or 1 × 10^6^ infected-erythrocytes (n = 2/group) on GD13.5, and the percentage of vaginally born dead fetuses was recorded. Mice exposed to 1 × 10^5^ infected-erythrocytes did not exhibit any signs of being born vaginally prior to GD18.5. On the other hand, the percentage of vaginally-born dead fetuses, among mice that received 5 × 10^5^ infected erythrocytes, was 12.5%, and, among the animals which received 1 × 10^6^ infected erythrocytes, this percentage was 100% (Supplementary Table 1). This pilot experiment was primarily conducted to obtain an effective dose and a point at which to start the establishment of a malaria induced-preterm labor model. Thus, we expanded this preliminary finding and administered 5 × 10^5^ infected erythrocytes (n = 20) or phosphate buffered saline (PBS - control; n = 12) on GD13.5, for posterior morphological analyses and protein/gene expression studies.

As shown in Table 1, pregnant malaria-infected mice (5 × 10^5^ infected erythrocytes) displayed reduced body weight gain between GD 13.5 and 18.5, and increased spleen weight, when compared to the control group (*P* = 0.011 and *P* < 0.001, respectively). However, there were no observable differences in litter size or fetal death rate between the two groups. Additionally, the results indicated that our model of gestational malaria promoted PTD, since 4 out of 20 females (20%) infected with *Plasmodium berghei ANKA* went into PTD on GD 17.5. i.e., exhibited visual signs of vaginal delivery on GD17.5. It was not possible to collect the placentae and fetuses from preterm deliveries that occurred on GD 17.5. As such, all analysis were performed on specimens collected on the day of euthanasia, GD 18.5, a gestational period when fetal growth, placental volume and maternal and fetal blood vessel surface areas peaks^23^. Offspring from malaria-infected mothers displayed reduced birth weight (*P* = 0.001) and fetal:placental (F:P) weight ratios (*P* = 0.002), with no significant changes in placental weight (Table 1). The average of peripheral parasitemia in the *Plasmodium*-infected group obtained on day GD18.5, immediately before euthanasia, was 16% of infected erythrocytes.

**Table 1:** Summary of pregnancy characteristics in *Plasmodium berghei Anka* infected mice and control mice at GD18.5.

	Mice (n)	Maternal weight gain (g)	Maternal spleen weight (mg)	Litter size	% Fetal death	% PTD	Placental weight (mg)	Fetal weight (mg)	F:P weight ratio
Control	12	6.5 ± 0.3	79.8 ± 3.4	5.4 ± 0.8	0 (0/80)	0 (0/12)	82.2 ± 0.8	1123 ± 47.5	13.6 ± 0.6
Malaria	20	3.78 ± 0.8^*^	153.8 ± 10.4^****^	5.7 ± 1.2	1 (1/99)	20 (4/20)	83.4 ± 0.1	843.9 ± 55.85^**^	9.71 ± 0.84^**^

Values are expressed as the mean ± SEM. *P < 0.05, **P < 0.01 and ****P < 0.0001.

### Pregnancy-associated malaria causes an intense systemic inflammatory response

**Figure 1:**
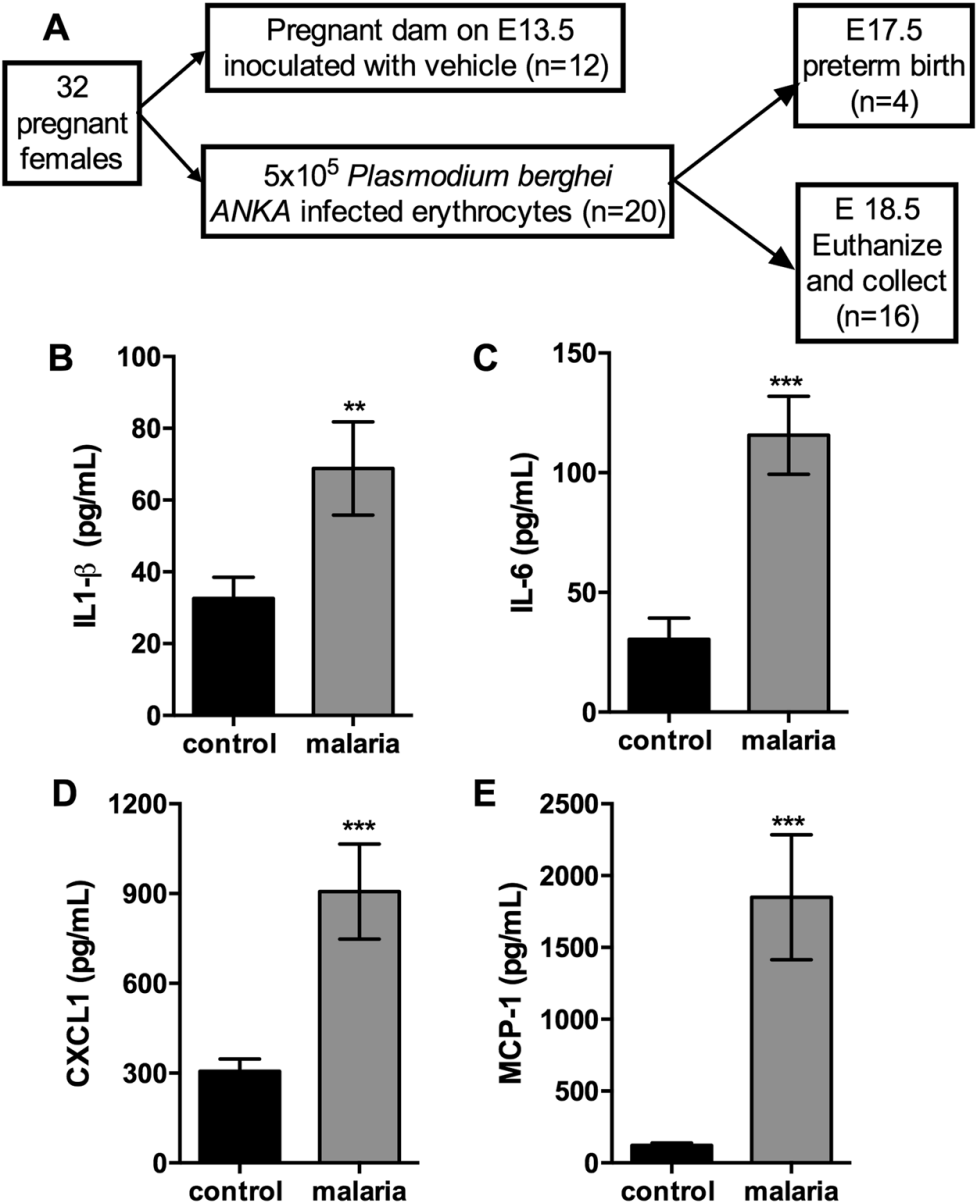
*Plasmodium berghei Anka* infection during pregnancy elicited an intense maternal inflammatory response. (**A**) Chart of the experimental design. Measurement of specific cytokine and chemokine contents in the maternal serum from pregnant mice infected with *Plasmodium berghei ANKA* on GD18.5. (**B**) IL1-β (n = 10/8); (**C**) IL-6 (n = 9/10); (**D**) CXCL1 (n = 10/10); (**E**) MCP-1 (n = 10/10). Values are expressed as the mean ± standard error. The unpaired Student’s *t* test was employed the for IL1-β, IL-6 and MCP-1 comparisons, and the Mann-Whitney test was used for comparing CXCL1 values.

Serum levels of IL1-β, IL-6, CXCL1 and MCP-1 were evaluated to determine whether gestational malaria induced a systemic inflammatory response. As shown in Fig. 1, there was an associated increase in IL1-β (Fig. 1B, *P* = 0.03), IL-6 (Fig. 1C, *P* = 0.001), CXCL1 (Fig. 1D, *P* = 0.001) and MCP-1 (Fig. 1E, *P* = 0.003) serum levels in malaria-infected mice, when compared to the control group, on GD18.5.

### Pregnancy-associated malaria induces specific placental ultrastructural alterations

**Figure 2:**
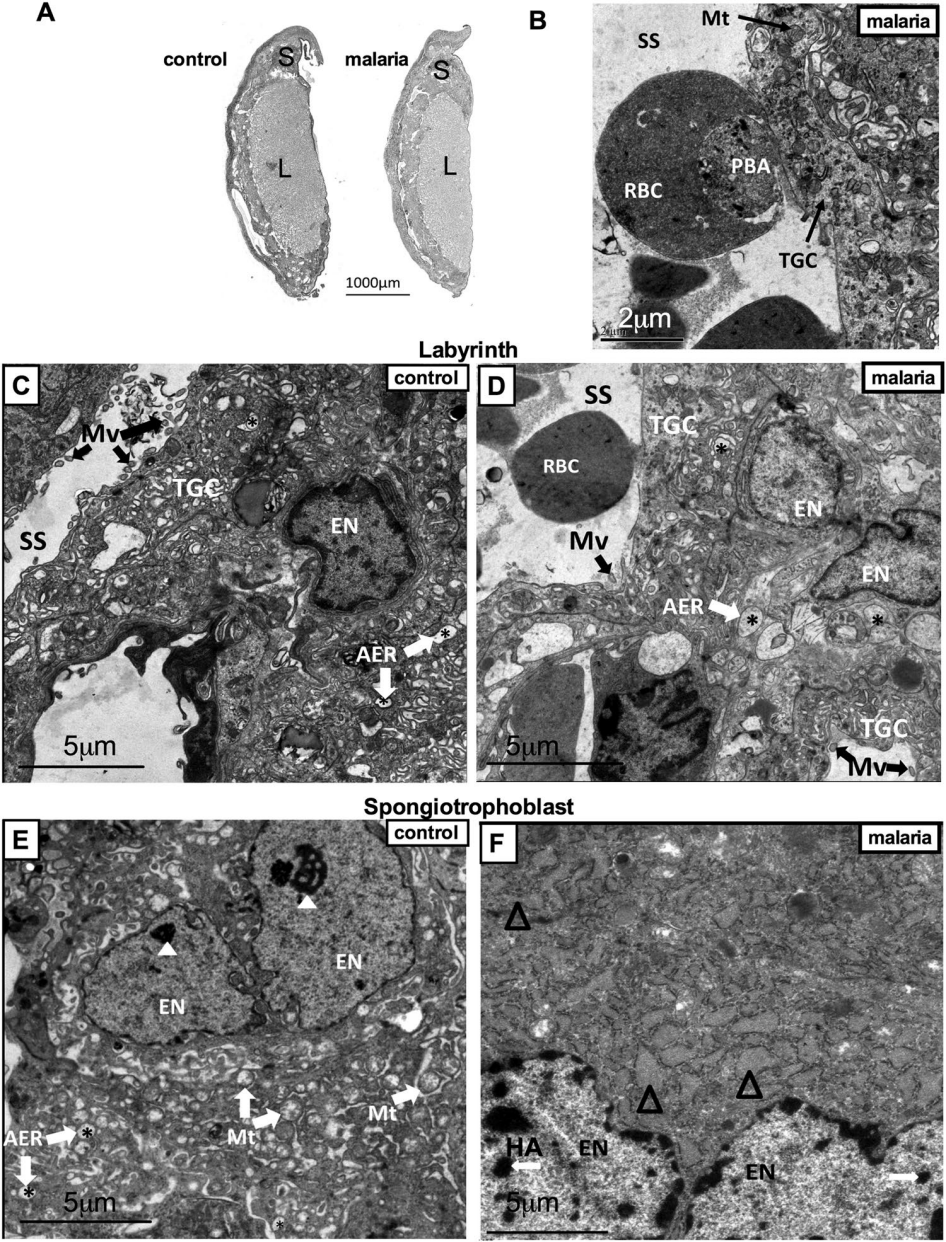
Evidence of *Plasmodium berghei ANKA-*infected erythrocyte accumulation and associated ultrastructural alterations in the placental labyrinth and spongiotrophoblast zones. (**A**) Placental gross morphological analysis. Periodic acid-Schiff staining of control and malaria-infected mice, evaluating total placental, labyrinth (L) and spongiotrophoblast (S) areas. (**B**) Transmission electron photomicrograph of malaria-infected erythrocyte adhering to labyrinthine trophoblast giant cell (TGC). (**C**,**D**) Transmission electron photomicrographs of the placental labyrinth. (**D**,**E**) and spongiotrophoblast areas. AER = *agranular endoplasmic reticulum; Δ dilated granular endoplasmic reticulum; Mt = mitochondria; Mv = microvilli; EN = euchromatic nucleus; PBA = *Plasmodium berghei ANKA;* RBC = red blood cell; SS = sinusoidal space; TGC = trophoblast giant cell; White arrows HA = heterochromatin area; White arrow heads = nucleolus. Scale bar = 2 µm (2B); 5 µm (2C-F).

Since gestational malaria, induced by *Plasmodium berguei ANKA* infection, reduced the F:P ratio, we analyzed the relative proportions of labyrinth and spongiotrophoblast areas, using PAS staining. There were no alterations in total placental, labyrinth and spongiotrophoblast areas, indicating the absence of gross morphological change (Fig. 2A). Evidence of placental accumulation of *Plasmodium*-infected erythrocytes was assessed using TEM (Fig. 2B), and revealed that infected erythrocytes were present in the labyrinthine sinusoids and were adhering to the labyrinthine interhemal membrane exchange site, which is consistent with previous work^24^. Ultrastructural analyses, using TEM, indicated that the labyrinth area (Fig. 2C) of control placentae was enriched with euchromatic nuclei and to a lesser extent, heterochromatin regions. Additionally, microvilli in sinusoidal trophoblastic giant cells and large agranular endoplasmic reticulum were observed. Conversely, malaria-infected placentae (Fig. 2D), despite exhibiting large agranular endoplasmic reticulum, lacked or displayed fewer microvilli in the sinusoidal trophoblastic giant cells. Furthermore, the spongiotrophoblasts from control placentae (Fig. 2E) exhibited euchromatic nuclei, evident nucleoli, substantial mitochondrial content and large agranular endoplasmic reticulum. In contrast, malaria-infected placentae (Fig. 2F) contained a greater number of heterochromatic regions and contained granular endoplasmic reticulum with dilated cisterns.

### Placental malaria increases cell proliferation without altering the apoptotic index

**Figure 3:**
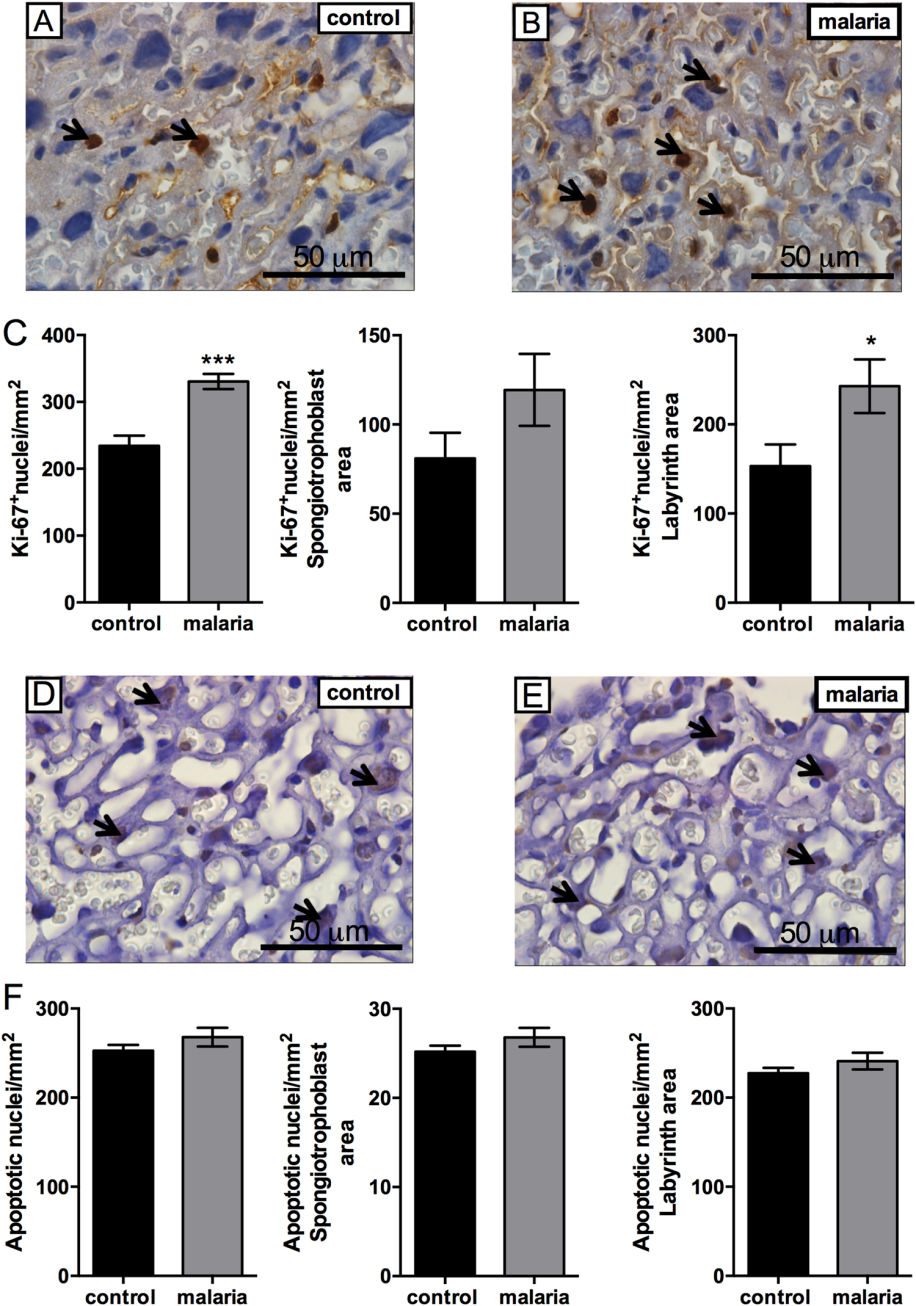
Placental malaria induces hyperproliferation in the labyrinth zone. (**A**) Representative immunohistochemistry images of Ki-67 positive cell nuclei in control and (**B**) pregnant malaria infected mice. (**C**) Graphs represent Ki-67 counting in the total placental, spongiotrophoblast and labyrinth area, respectively. (**D**) Immunohistochemical staining for apoptotic nuclei detection, using the TUNEL method, in the placenta of control and (**E**) malaria-infected mice. (**F**) Graphs show the apoptotic cell nuclei counting in the total placental, spongiotrophoblast and labyrinth areas, respectively. Black arrows = Ki-67/TUNEL stained nuclei. Values are expressed as the mean ± SEM. The labyrinth, spongiotrophoblast and total placental area values, in both Ki-67 and TUNEL experiments were analyzed using the unpaired Students T-test. N = 5/group. Scale bar = 50 μm.

Since we did not observe changes in placental weight following *Plasmodium berghei ANKA* infection, we investigated whether proliferation or apoptotic rates were altered in gestational malaria. Using the Ki-67 proliferation marker^25^, immunohistochemistry analysis revealed an increase in total placental cell proliferation (Fig. 3C, *P* = 0.001), which was more evident in the labyrinth (Fig. 3C, *P* = 0.050) than the spongiotrophoblast zone of the murine placenta. Conversely, TUNEL analysis did not reveal any significant differences in the apoptotic index in the total placental area or in the labyrinth/spongiotrophoblast zones (Fig. 3D–F).

### Pregnancy-associated malaria induces placental and maternal hepatic inflammatory responses and impairs ABC transporter gene expression

**Figure 4:**
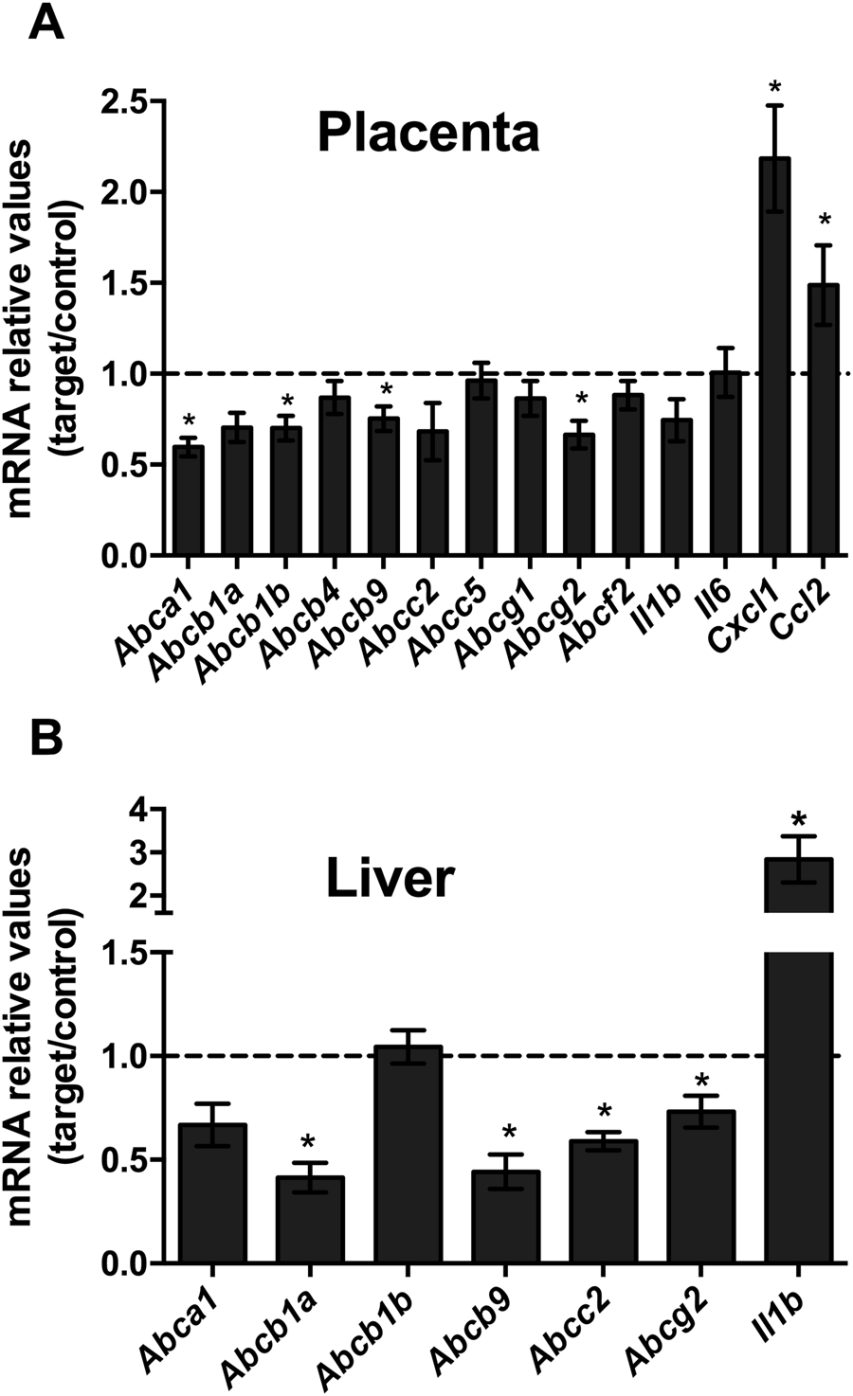
Pregnancy associated malaria induces an inflammatory response in the placenta and maternal liver and downregulates the expression of key ABC transporter genes. (**A**) Placental levels of *Abca1*, *Abcb1a*, *Abcb1b*, *Abcb4*, *Abcb9*, *Abcc2*, *Abcc5*, *Abcg1*, *Abcg2*, *Abcf2*, *Il6*, *Cxcl1*, *Ccl2* and *Il1b* mRNA (n = 12/16). (**B**) Levels of *Abca1*, *Abcb1a*, *Abcb1b*, *Abcb9*, *Abcc2*, *Abcg2* and *Il1b* mRNA in the maternal liver (n = 12/14). The broken line indicates the expression levels in the control group which were set to one. Values are expressed as the mean ± SEM. *P < 0.05. Unpaired Students t-tests were undertaken in both the placenta and maternal liver analysis.

The expression of *Abca1*, *Abcb1b*, *Abcb9 and Abcg2* was downregulated in the placentae of *Plasmodium berghei ANKA*-infected mice (Fig. 4A, *P* < 0.05), whereas there was a 2-fold increase in *Cxcl1* and a 1.4-fold increase in *Ccl2* mRNA expression, when compared to controls (Fig. 4A, *P* < 0.05). In order to determine if the effects of PAM on ABC transporters regulation were tissue specific, the expression of key ABC transporters was also assessed in the maternal liver. Hepatic levels of *Abcb1a*, *Abcb9*, *Abcc2* and *Abcg2* mRNA were reduced (Fig. 4B, *P* < 0.05), however, the expression of *Abca1* and *Abcb1b* were not affected by PAM. In addition, *Il1b* expression in the liver was upregulated in the malaria-infected group, when compared to controls (Fig. 4B, *P* < 0.05).

### Pregnancy-associated malaria impairs protein expression of major ABC transporters involved in cholesterol transfer and fetal protection

**Figure 5:**
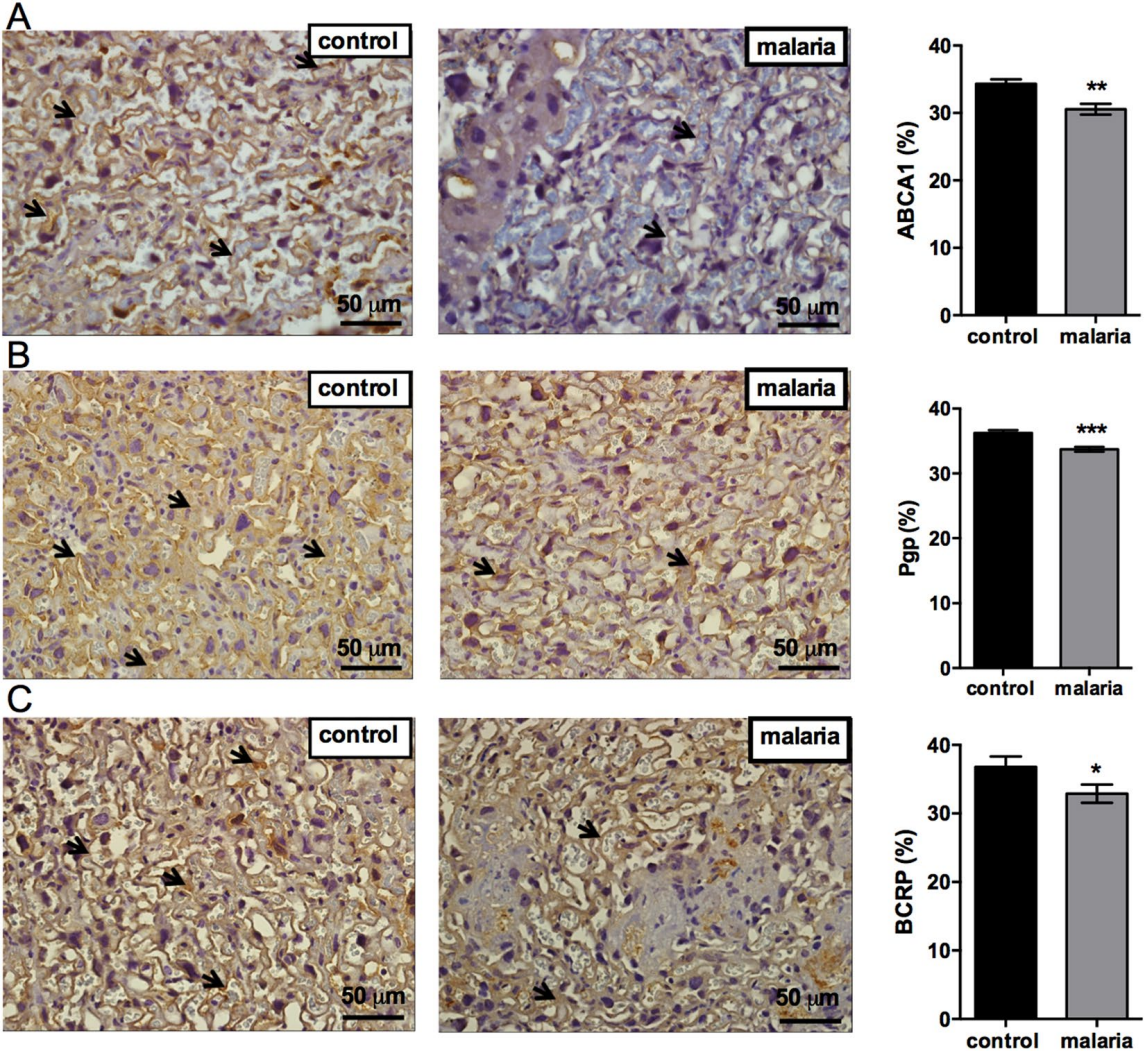
Placental malaria decreases ABCA1, P-glycoprotein and breast cancer resistance protein expression in the placenta. Representative immunohistochemistry images and immunostaining quantification of (**A**) ABCA1; (**B**) P-gp; and (**C**) BCRP in the placenta of control and malaria-infected mice. The black arrows represent ABCA1/P-gp/BCRP stained cells. Graphs represents the % of stained cells/viable tissue. Values are expressed as the mean ± SEM. The unpaired Student’s *t* test was used. N = 5. *P < 0.05, **P < 0.004. Scale bar = 50 μm.

Due to the fact that *Abca1*, *Abcb1b* and *Abcg2* were downregulated in malaria infected pregnancies, the protein expression levels of the correspondent best described ABC transporters, ABCA1, P-gp and BCRP were assessed. Consistent with the gene expression responses, the levels of ABCA1, P-gp and BCRP protein were significantly reduced in the malaria infected animals, when compared to controls (Fig. 5A–C).

## Discussion

In this study, we have developed a new murine model of MiP that recapitulates many of the features associated with human placental malaria^26^. For example, the accumulation of *Plasmodium*-infected erythrocytes into the labyrinthine sinusoids, IUGR and PTD were all observed in this animal model. There were also intense maternal systemic and placental inflammatory responses. Placental ultrastructural analyses of labyrinthine cells revealed a loss of microvilli in sinusoidal trophoblastic giant cells, whereas spongiotrophoblasts exhibited a greater number of heterochromatic regions and granular endoplasmic reticulum with dilated cisterns. Infected mice exhibited reduced placental levels of *Abca1*, *Abcb1b*, *Abcg2* and *Abcb9* mRNA, which were accompanied by reductions in placental ABCA1, BCRP and P-gp protein.

Using C57BL/6 mice infected with *P*. *berghei ANKA*, we were able to induce placental malaria, IUGR and PTD. Typically, this type of infection is used to induce cerebral malaria, which is a more severe form of the disease^19,27^. Furthermore, C57BL/6 mice are particularly more susceptible to *P*. *berghei ANKA* infection compared to the BALB/c mice^19^. This likely account for the more severe phenotype observed in pregnant C57BL/6 mice, compared to pregnant BALB/c mice infected with *P*. *berghei ANKA*^12,20^. However, it is important to note that pregnant BALB/c mice infected with *P*. *berghei ANKA* also exhibit IUGR, syncytiotrophoblast damage, placental parasite accumulation and inflammation, as well as, increased maternal spleen weight and decreased maternal weight^28,29^. In this context, the average parasitemia observed on GD18.5 in C57BL/6 *Plasmodium*-infected mice (16% of infected erythrocytes) is consistent with the average observed in *Plasmodium*-infected BALB/c mice^20^, suggesting that this range of parasitemia is consistently, associated with trophoblast disruption in different experimental murine malaria models.

The dose of *P*. *berghei ANKA* (5 × 10^5^) used following pilot studies, induced PTD in 20% of the mice, promoted IUGR and reduced the F:P weight ratio. The latter is an indirect measure of placental efficiency^30^, indicating that placentae from malaria-infected mice are less efficient, and likely accounting for the reduced fetal weight observed. Use of TEM, allowed us to detect less sinusoidal trophoblastic giant cells -microvilli in the labyrinth of malaria-infected placentae, indicating impaired placental uptake of nutrients from the maternal blood and possibly explaining, at least in part, the decreased placental efficiency. In parallel, malaria infection compromised maternal weight gain, which might well contribute to the observed IUGR phenotype in this model.

One of the hallmarks of human placental pathogenesis in malaria is the accumulation of *Plasmodium*-infected erythrocytes into the intervillous space of the placenta, which adhere to specific glycosaminoglycans expressed in the syncytiotrophoblasts^31,32^. Here, we provide evidence that *P*. *berghei ANKA* infected erythrocytes were accumulated into the labyrinthine sinusoid space of infected mice, which is bathed in maternal blood. Accumulation of infected-erythrocytes was also described in a BALB/c model of MiP^20^, thus demonstrating that this pathogen has the ability to induce placental malaria in different mouse strains. In addition, other ultrastructural changes were detected in the spongiotrophoblast zone of infected animals. These include the presence of well-developed granular endoplasmic reticulum with dilated cisterns. These findings are indicative of increased protein accumulation in the placenta, and could be related to the increase in labyrinthine cell proliferation. It is also possible that hyperproliferation of the labyrinth zone is a placental adaptation necessary for meeting the fetal growth requirements in this severe pathological state, without altering placental weight, the apoptotic ratio or gross morphology. Alternatively, it may be a direct response to placental inflammation, triggered by the presence of infected erythrocytes in the sinusoidal space of the placenta.

The upregulation in placental *Cxcl1* mRNA expression, a human IL-8 analog, is another important finding, and may play a role in the increased PTD rate, since this cytokine has been directly linked to the inflammatory milieu typically found in pregnancies with a heightened risk for PTD^33^. Furthermore, the increase in *Il1-b* mRNA expression in the maternal liver, not only serves as an indicator of systemic inflammation, but may also be linked to the downregulation of specific ABC transporter expression in the placenta and maternal liver^34^. Furthermore, increased maternal plasma cytokine levels, especially IL1-*β* and IL-6, are established markers of the inflammatory response responsible for cervical ripening and PTD induction^35^.

Following the development of a MiP model that recapitulates many of the features of the disease in humans, we set out to investigate the placental expression of selected ABC transporters, which are related to nutrient transfer and fetal protection against drugs and environmental toxins. Among these transporters, it was found, for the first time, that both the gene and protein expression levels of Abca1 were downregulated in placental malaria. In humans, this protein is predominantly expressed in the apical membrane of syncytiotrophoblasts, and functions to transport cholesterol, phospholipids and cytotoxic oxysterols out of the cell and into the maternal circulation^36^. Importantly, ABCA1 also extrudes inflammatory mediators, such as the macrophage migration inhibitory factor (MIF) which are associated with inflammatory processes involved in the onset of PTD^37^. Impaired placental expression of Abca1, in placental malaria, may result in compromised lipid homeostasis, as evidenced by the presence of well-developed agranular endoplasmic reticulum with dilated cisterns in labyrinthine cells. It may also result in accumulation of cytotoxic oxysterols and/or MIF in the fetal compartment. Placental *Abcb9* mRNA expression was also downregulated in malaria-infected mice; however, whether this decrease is related to malarial infection that leads to PTD, remains to be determined; its location in the placental barrier is unknown. Notwithstanding, this protein has been shown to be involved in inflammatory responses associated with T lymphocytes^38^ and expression of its mRNA is disrupted in human preterm placentas with chorioamnionitis^18^.

Placental malaria decreased *Abcb1b* and *Abcg2* mRNA and their protein products P-gp and BCRP. These proteins represent major multidrug resistance transporters, and a reduction in placental expression is consistent with previous findings demonstrating that bacterial and viral challenges can decrease human placental P-gp and BCRP expression and murine placental P-gp activity^15–18^. These findings are also consistent with a previous study from Cressman *et al*., who demonstrated that *Plasmodium berghei ANKA*-infected BALB/c mice exhibited reduced placental *Abcb1b* and *Abcg2* mRNA expression and decreased P-gp protein expression. On the other hand, contrary to our findings, Cressman *et al*. also demonstrated a reduction in the placental expressions of *Abcb1a* and *Abcc2* and an increase in the hepatic expression of *Abcb1b*, which can be explained by the use of different mouse strains and experimental designs^12^. However, it remained to be determined whether placental reduction of these multidrug resistance transporters was associated with the pathogenesis of malarial-induced PTD and related IUGR. In this connection, here we show for the first time a reduction in placental P-gp and BCRP expression could be potentially involved in the mechanisms of malarial induced PTD and IUGR. Importantly, P-gp and BCRP have been related to the pathogenesis of PTD induced by chorioamnionitis^18^, whereas impaired P-gp expression has been demonstrated in placentas from growth restricted preterm fetuses^39^. Moreover, P-gp and BCRP are enriched in the apical membrane of syncytiotrophoblasts and transport their substrates from fetal to maternal circulation. Impaired placental expression of these multidrug resistance transporters during MiP, could potentially augment the fetal accumulation of drugs commonly prescribed during pregnancy, including: antibiotics, antihistamines, antiretrovirals, NSAIDs and others^13^, as well as increase fetal exposure to different environmental toxicants. Such an accumulation could negatively impact fetal outcome, gestational length or influence the dose requirements of these medications in pregnant malaria-infected individuals. These possibilities, however, require further investigation given that reduction in ABCA1, P-gp and BCRP expression, while significant, were of only moderate magnitude.

In the maternal liver, there was a decrease in *Abcb1a*, *Abcc2*, *Abcb9* and *Abcg2* gene expression, whereas in the placenta, we observed a decrease in *Abca1*, *Abcb1b*, *Abcb9* and *Abcg2*. With the exception of *Abcb1b*, a similar pattern was also observed in *Plasmodium berghei* ANKA-infected BALB/c mice^12^, which may indicate that gestational malaria elicits similar regulatory pathways for the expression of ABC transporters in different tissues. Importantly, expression of *Abcc2*, a transporter related to bile salt excretion in the liver, was assessed as dysregulation of bile salts in the maternal serum has been linked to PTD^12^.

Limitations of the present model include our inability to dissociate effects from experimental cerebral malaria (ECM) and effects associated with the onset of PTD. However, it is important to note that mice were closely monitored daily between GD 13.5 and GD 18.5 and behavior associated with ECM including ataxia and paralysis was evident from GD17.5. Suggesting that consequences of ECM may be associated with the phenotype herein observed.

In conclusion, the present study describes a new model of pregnancy-associated malaria using C57BL/6 mice infected with *Plasmodium berghei ANKA*. This model recapitulates features commonly observed in human MiP, including IUGR and PTD. Based on the results, IUGR may result from the loss of labyrinthine-microvilli, as well as the downregulation of selected ABC transporters and other placental nutrient and drug transporters previously demonstrated to be altered in human and experimental MiP. However, this is the first time that dysregulation of placental ABC transporters has been related to malaria induced-preterm labor. These alterations are probably due to the sequestration of malaria-infected erythrocytes into the placental sinusoidal space, leading to an intense pro-inflammatory response, potentially resulting in increased levels of harmful physiological and pharmacological factors in the placenta and fetal circulation.

## Materials and Methods

### Animal experimentation and Study Design

Mice used in this study were housed in a temperature-controlled room (23 °C), under a 12/12 h light/dark cycle, and had free access to fresh food and water. In total, 32 virgin female C57BL/6 mice (8–10 weeks of age) were bred with 10 C57BL/6 males, as previously described^15^. The following morning, the female mice were examined for vaginal plugs. If present, female mice were considered to be at gestational day 0.5 (GD0.5). This study was approved by the Animal Care Committee of the Health Sciences Center, Federal University of Rio de Janeiro (CEUA-190/13) and registered with the Brazilian National Council for Animal Experimentation Control. The animal’s human humane cares were in compliance with the “Principles of Laboratory Animal Care” formulated by the National Society for Medical Research and the U.S. National Academy of Sciences Guide for the Care and Use of Laboratory Animals.

On GD13.5, shortly after the complete formation of murine placental circulation^40^, animals were injected intraperitoneally (i.p). with a single dose of *Plasmodium berghei ANKA-*infected erythrocytes (5 × 10^5^ infected-erythrocytes, n = 20) or phosphate buffered saline (PBS - control; n = 12), as described previously^20,41^, with modifications. The murine *Plasmodium berghei ANKA* model of malaria infection was chosen because it recapitulates many features of pregnancy-associated malaria^20^. In the present study, we have adapted its use to generate a new murine model of malaria-induced PTD, associated with IUGR. Briefly, murine infection was achieved with infected erythrocytes, obtained from animals exposed to cryopreserved protozoa. Pilot studies were conducted to determine the appropriate infected-erythrocyte regimen necessary for eliciting PTD in 10–20% of the animals, on GD17.5. This stage of gestation was selected based on evidence that murine term birth occurs between GD18 and 22, with an average gestational period of 19.25 days for C57BL/6 mice^21^.

On GD18.5, maternal parasitemia was determined using Giemsa-stained thick blood smears obtained from caudal vein, before euthanasia. The percentage of infected erythrocytes was defined as the number of erythrocytes infected per 100 erythrocytes counted in ten fields, using light microscopy.

On the morning of GD18.5, both groups were anesthetized with isoflurane (Cristalia, São Paulo, Brazil), and euthanized by decapitation. Maternal plasma was harvested by cardiac puncture, placed into heparinized tubes on ice, immediately centrifuged (1,077 × g, 15 min) and frozen at −80 °C until the analysis of cytokine levels. The maternal liver was dissected and preserved in RNAlater stabilizing solution (Thermo Fisher Scientific, Massachusetts, USA), and stored at −70 °C until qPCR analysis. The weight of the maternal spleen was also recorded.

Following decapitation, the fetuses and placental discs were dissected and weigthed. Two placentae, with wet weights closest to the mean weight of all placentae, were selected from each litter^42^ for qPCR analysis, or incubated in 4% buffered paraformaldehyde (Sigma-Aldrich, Missouri, USA) for morphological evaluation and assessment of protein levels and localization.

### qPCR

Total RNA from the maternal liver and placental discs was extracted using the TRIzol method according to the manufacturer’s instructions (TRIzol Reagent; Life Technologies, California, USA). RNA concentration and purity were assessed spectrophotometrically by measuring the absorbance values at 260 nm and the A260/A280 respectively, whereas RNA integrity was confirmed by gel electrophoresis. Total RNA (1 μg) was used to synthesize cDNA using the High Capacity cDNA Reverse Transcription Kit (Applied Biosystems, São Paulo, Brazil) according to the manufacturer’s instructions. The mRNA levels of selected ABC transporters and pro-inflammatory cytokines and chemokines (Table 2) were measured by qPCR using the Maxima SYBR Green/ROX qPCR Master Mix 2 × (Thermo Fisher Scientific, Massachusetts, USA) and Master Cycler Realplex system (Eppendorf, Germany), with the following cycling conditions: combined initial denaturation at 50 °C (2 min) and 95 °C (10 min), followed by 40 cycles of denaturation at 95 °C (15 s), annealing at 60 °C (30 s) and extension at 72 °C (45 s). Gene expression was normalized to the geometric mean of reference genes, *Gapdh* and *Ywhaz*, which exhibited stable expression levels following *Plasmodium berghei ANKA* infection (Table 2). Relative gene expression was calculated according to the 2^−ΔΔCT^ method^43^. The assay was considered acceptable when its efficiency ranged from 95 to 105%. DNA contamination was ruled out using intron-spanning primers (Table 2), reverse transcriptase-negative samples and melting curve analyses obtained from each qPCR reaction. All samples and standards were measured in duplicate.

**Table 2:** Primers Used in The Present Study.

Primers	Sequence	Reference	Genbank accession no.
*Abca1*	5′GCAGATCAAGCATCCCAACT 3′ 3′CCAGAGAATGTTTCATTGTCCA 5′	^48^	NM_013454.3
*Abcb1a*	5′GGGCATTTACTTCAAACTTGT 3′ 3′TTTACAAGCTTCATTTCTCAA 5′	^48^	NM_011076.3
*Abcb1b*	5′AAGCCAGTATTCTGCCAAGCAT 3′ 3′CTCCAGACTGCTGTTGCTGATG 5′	^48^	NM_011075.2
*Abcb4*	5′GAAGGGATCTACTTCAGACTCGTT 3′ 3′TCAACTTCAAATTCTTCTGACAGG 5′	^48^	NM_008830.2
*Abcb9*	5′GTGTATTGTTGGATGGCAAGC 3′ 3′GGAGATGTTGTCTGTGATGGAG 5′	^49^	NM_019875.2
*Abcc2*	5′TAATGAGGCGCCGTGGGTGAC 3′ 3′GTCCTGCCCACCACACCGAC 5′	^50^	NM_013806.2
*Abcc5*	5′AAATGTATGCCTGGGTCAAAGC3′ 3′TGGCGATCACTACCACAATAGG5′	*	NM_013790.2
*Abcg2*	5′TGCCAGGCGCTCATTTAAAAACTTGC3′ 3′GCATTCCAGCGGCATCATATTTCAGA 5′	^50^	NM_011920.3
*Abcf2*	5′TGTCCACATTATCAACCTCTCCC 3′ 3′TCACGTTTCCCAATAGCCGAG 5′	*	NM_013853.2
*Il6*	5′GAGGATACCACTCCCAACAGACC 3′ 3′AAGTGCATCATCGTTGTTCATACA 5′	^49^	NM_031168.2
*Cxcl1*	5′ACCCGCTCGCTTCTCTGT 3′ 3′AAGGGAGCTTCAGGGTCAAG 5′	^49^	NM_008176.3
*Ccl2*	5′GGTCCCTGTCATGCTTCTGG 3′ 3′CCTGCTGCTGGTGATCCTCT 5′	^51^	NM_011333.3
*Gapdh*	5′TGTGTCCGTCGTGGATCTGA 3′ 3′TTGCTGTTGAAGTCGCAGGAG 5′	^52^	NM_001289726.1
*Ywhaz*	5′ GAAAAGTTCTTGATCCCCAATGC 3′ 5′ TGTGACTGGTCCACAATTCCTT 3′	*	NM_011740.3
*β-actin*	5′ AAATCTGGCACCACACCTTC 3′ 5′ GGGGTGTTGAAGGTCTCAAA 3′	^53^	NM_007393.5
*Ppib*	5′ GAGACTTCACCAGGGG 3′ 5′ CTGTCTGTCTTGGTGCTCTCC 3′	*	NM_011149.2
*Rplp0*	5′ GGCCCTGCACTCTCGCTTTC 3′ 5′ TGCCAGGACGCGCTTGT 3′	*	NM_007475.5

^*^Gene specific primers were designed with primer-BLAST (http://www.ncbi.nlm.gov/tools/primer-blast).

### Histological analysis of the placenta, immunohistochemistry and TUNEL

Fixed placentae were dehydrated protocol with increasing concentrations of ethanol, diaphanization in xylol and inclusion in paraffin. The blocks were sectioned (5 μm), using a Rotatory Microtome CUT 5062 (Slee Medical GmbH, Germany), and sections subjected to Periodic Acid-Schiff (PAS) staining and immunohistochemistry. Following diaphanization with three xylene immersions, and hydration with decreasing concentrations of ethyl alcohol (100%, 90% and 70%), placental sections were oxidized with 0.5% periodic acid (Sigma-Aldrich, Missouri, USA) for 15 minutes, washed in distilled water and incubated with Schiff’s reagent (Merck, Germany; 10 min, room temperature). Subsequently, each section was washed with distilled water, stained with hematoxylin (Proquímios, Rio de Janeiro, Brazil), dehydrated with increasing concentrations of ethyl alcohol (70%, 90%, 100%), clarified in xylene and mounted with Entellan (Merck, Germany). The limit between the maternal and fetal components of the placenta was identified by the presence of giant trophoblastic cells (GTC), which separate the basal decidua from the junctional zone^40^, and the area of each region was measured using the free-drawing tool of the Image J software (National Institutes of Health, Maryland, USA).

For immunohistochemical analyses, following deparaffinization and rehydration, sections were exposed to hydrogen peroxide (3%) diluted in PBS. Excess peroxide was removed with PBS + Tween. Antigen retrieval was achieved by immersing the slides in Tris-EDTA buffer (pH 9.0), followed by immersion in sodium citrate buffer (pH 6.0), in the microwave (15 minutes for Tris-EDTA buffer and 8 minutes for citrate buffer). The slides were then incubated in bovine serum albumin (3%) in PBS, for 1 hour to block non-specific antibody binding. Slides were then incubated with primary antibodies for: Ki67 (1:100; Spring Bioscience, California, USA), P-gp (1:500; Santa Cruz Biotechnology, Texas, USA), BCRP (1:100; Merck Millipore, Massachusetts, USA) or ABCA1 (1:100; Abcam Plc, UK) overnight at 4 °C. The next day, the slides were incubated with biotin-conjugated secondary antibody (SPD-060 - Spring Bioscience, California, USA) for 1 hour. After incubation with streptavidin (SPD-060 - Spring Bioscience, California, USA) for 1 hour, the reaction was stopped with 3,3-diamino-benzidine (DAB) (SPD-060 - Spring Bioscience, California, USA). The terminal deoxynucleotidyl transferase dUTP nick end labeling (TUNEL) method was used for the detection of apoptotic nuclei, using the ApopTag® *In Situ* Peroxidase Detection Kit (Merck Millipore, Massachusetts, USA), according to the manufacturer’s recommendations.

After immunostaining and TUNEL, the sections were counterstained with hematoxylin, dehydrated in increasing concentrations of ethanol and immersed in xylol and mounted with laminula and Entellan (Merck, Germany). Sections were imaged, under bright-field illumination, with a high-resolution Olympus DP72 camera (Olympus Corporation, Japan) coupled to an Olympus BX53 light microscope (Olympus Corporation, Japan).

Quantification of Ki-67 and TUNEL immunostained nuclei was performed using the STEPanizer software^44^. Fifteen digital images were captured per tissue fragment of each placental zone (labyrinth and spongiotrophoblast) in 5 control mice and 5 malaria-infected mice, totaling 300 digital images analyzed: five animals with parasitemia value closest to the global parasitemia average in the MiP group, whereas control animals were randomly selected. The Ki-67 and TUNEL immunostained nuclei from each image were divided by the image area, which yielded an estimate for the number of proliferative or apoptotic nuclei present in the whole tissue^45^. Quantification of P-gp, BCRP and ABCA1 was performed with the mask tool present in the Image Pro Plus 5.0 software (Media Cybernetics, Maryland, USA), where only the percentage of viable tissue area was considered, and empty spaces of the images were excluded. A total of 30 digital images per placenta (15 digital images for each placental area, labyrinth and spongiotrophoblast) were evaluated in each experimental group.

### Transmission electron microscopy (TEM)

After euthanasia, placental fragments were fixed in glutaraldehyde (Sigma-Aldrich, Missouri, USA, 2.5%, 48 hours). The fragments were then immersed in cacodylate buffer (Sigma-Aldrich, Missouri, USA; 3 × 15 minutes). Samples were post-fixed (60 minutes) with osmium tetroxide and potassium ferrocyanide. The placental fragments were dehydrated in increasing concentrations of PA acetone (30%, 50%, 70%, 90% and 2 × 100%) and subjected to EPOXI resin infiltration using baths with increasing ratios of EPOXI resin to PA acetone (1:2, 1:1 and 2:1). After polymerization, semi-thin sections (2 µm) (Leica Microsystems, Germany) were produced. To select the region to be analyzed, ultra-thin sections (70 nm) (Leica Microsystems, Germany) were used, which were further contrasted with uranyl acetate and lead citrate and later visualized using a JEM-1011 transmission electron microscope (JEOL Ltd., Japan).

### Measurement of Serum Cytokine and Chemokine levels

Maternal serum interleukin (IL)-6, IL1-β, monocyte chemoattractant protein-1 (MCP-1/CCL2) and the chemokine (C-X-C motif) ligand 1 (CXCL1) concentrations were measured using the commercially available MILLIPLEX-MAP Mouse Cytokine/Chemokine Magnetic Bead Panel – Immunology Multiplex Assays (Merck Millipore, Massachusetts, USA), according to manufacturer’s protocol recommendations, and fluorescence intensity was detected using a Luminex 200™ system (Merck Millipore, Massachusetts, USA).

### Statistical analysis

All data are expressed as the mean ± standard error of the mean (SEM). Normality tests were applied to evaluate normal distribution. The Student’s T-test or non-parametric Mann-Whitney test were applied accordingly when performing comparisons between the two groups. Statistical assessment of pregnancy parameters, were undertaken using the mean value of all fetuses in a litter for each C57BL/6 mother, and not the individual fetuses. For qPCR and immunostaining data, closest placentae to the mean weight of all placentae were selected from each litter. Thus “n” represents the number of litters^42,46,47^. Statistical analyses were performed using the Graphpad prism 6 software (GraphPad, Inc., California, USA) and differences were considered to be significant at *P* < 0.05.

## Supplementary information

Sypplementary table 1

## References

[1] Goldenberg Robert L, Culhane Jennifer F, Iams Jay D, Romero Roberto (2008). Epidemiology and causes of preterm birth. The Lancet.

[2] Chawanpaiboon Saifon, Vogel Joshua P, Moller Ann-Beth, Lumbiganon Pisake, Petzold Max, Hogan Daniel, Landoulsi Sihem, Jampathong Nampet, Kongwattanakul Kiattisak, Laopaiboon Malinee, Lewis Cameron, Rattanakanokchai Siwanon, Teng Ditza N, Thinkhamrop Jadsada, Watananirun Kanokwaroon, Zhang Jun, Zhou Wei, Gülmezoglu A Metin (2019). Global, regional, and national estimates of levels of preterm birth in 2014: a systematic review and modelling analysis. The Lancet Global Health.

[3] Passini Renato, Cecatti Jose G., Lajos Giuliane J., Tedesco Ricardo P., Nomura Marcelo L., Dias Tabata Z., Haddad Samira M., Rehder Patricia M., Pacagnella Rodolfo C., Costa Maria L., Sousa Maria H. (2014). Brazilian Multicentre Study on Preterm Birth (EMIP): Prevalence and Factors Associated with Spontaneous Preterm Birth. PLoS ONE.

[4] Lettieri Luanna, Vintzileos Anthony M., Rodis John F., Albini S. Mark, Salafia Carolyn M. (1993). Does “idiopathic” preterm labor resulting in preterm birth exist?. American Journal of Obstetrics and Gynecology.

[5] Challis John R., Lockwood Charles J., Myatt Leslie, Norman Jane E., Strauss Jerome F., Petraglia Felice (2009). Inflammation and Pregnancy. Reproductive Sciences.

[6] Ghazanfari, N., Mueller, S. N. & Heath, W. R. Cerebral Malaria in Mouse and Man. *Front*. *Immunol*, 10.3389/fimmu.2018.02016 (2018). 10.3389/fimmu.2018.02016 PMC6139318 30250468

[7] Sharma, L. & Shukla, G. Placental Malaria: A New Insight into the Pathophysiology. *Front*. *Med*, 10.3389/fmed.2017.00117 (2017). 10.3389/fmed.2017.00117 PMC5524764 28791290

[8] Bauserman Melissa, Conroy Andrea L., North Krysten, Patterson Jackie, Bose Carl, Meshnick Steve (2019). An overview of malaria in pregnancy. Seminars in Perinatology.

[9] Souza Rodrigo M., Ataíde Ricardo, Dombrowski Jamille G., Ippólito Vanessa, Aitken Elizabeth H., Valle Suiane N., Álvarez José M., Epiphânio Sabrina, Marinho Claudio R. F. (2013). Placental Histopathological Changes Associated with Plasmodium vivax Infection during Pregnancy. PLoS Neglected Tropical Diseases.

[10] Boeuf Philippe, Aitken Elizabeth H., Chandrasiri Upeksha, Chua Caroline Lin Lin, McInerney Bernie, McQuade Leon, Duffy Michael, Molyneux Malcolm, Brown Graham, Glazier Jocelyn, Rogerson Stephen J. (2013). Plasmodium falciparum Malaria Elicits Inflammatory Responses that Dysregulate Placental Amino Acid Transport. PLoS Pathogens.

[11] Najjar Najwa, McColl Eliza R., Weckman Andrea, Kain Kevin C., Piquette‐Miller Micheline (2019). Dysregulation of solute carrier transporters in malaria‐infected pregnant mice. Parasite Immunology.

[12] Cressman A. M., McDonald C. R., Silver K., Kain K. C., Piquette-Miller M. (2013). Malaria Infection Alters the Expression of Hepatobiliary and Placental Drug Transporters in Pregnant Mice. Drug Metabolism and Disposition.

[13] Bloise, E. *et al*. ATP-binding cassette transporters in reproduction: A new frontier. *Human Reproduction Update*, 10.1093/humupd/dmv049 (2016). 10.1093/humupd/dmv049 PMC4755438 26545808

[14] Imperio GE (2019). Gestational age‐dependent gene expression profiling of ATP‐binding cassette transporters in the healthy human placenta. J. Cell. Mol. Med..

[15] Bloise Enrrico, Bhuiyan Manzerul, Audette Melanie C., Petropoulos Sophie, Javam Mohsen, Gibb William, Matthews Stephen G. (2013). Prenatal Endotoxemia and Placental Drug Transport in The Mouse: Placental Size-Specific Effects. PLoS ONE.

[16] Lye Phetcharawan, Bloise Enrrico, Javam Mohsen, Gibb William, Lye Stephen J., Matthews Stephen G. (2015). Impact of Bacterial and Viral Challenge on Multidrug Resistance in First- and Third-Trimester Human Placenta. The American Journal of Pathology.

[17] Bloise Enrrico, Petropoulos Sophie, Iqbal Majid, Kostaki Alisa, Ortiga-Carvalho Tania Maria, Gibb William, Matthews Stephen G. (2017). Acute Effects of Viral Exposure on P-Glycoprotein Function in the Mouse Fetal Blood-Brain Barrier. Cellular Physiology and Biochemistry.

[18] do Imperio Guinever Eustaquio, Bloise Enrrico, Javam Mohsen, Lye Phetcharawan, Constantinof Andrea, Dunk Caroline, dos Reis Fernando Marcos, Lye Stephen James, Gibb William, Ortiga-Carvalho Tania M., Matthews Stephen Giles (2018). Chorioamnionitis Induces a Specific Signature of Placental ABC Transporters Associated with an Increase of miR-331-5p in the Human Preterm Placenta. Cellular Physiology and Biochemistry.

[19] de Oca Marcela Montes, Engwerda Christian, Haque Ashraful (2013). Plasmodium berghei ANKA (PbA) Infection of C57BL/6J Mice: A Model of Severe Malaria. Mouse Models of Innate Immunity.

[20] Neres Rita, Marinho Claudio R. F., Gonçalves Lígia A., Catarino Manuela Beirão, Penha-Gonçalves Carlos (2008). Pregnancy Outcome and Placenta Pathology in Plasmodium berghei ANKA Infected Mice Reproduce the Pathogenesis of Severe Malaria in Pregnant Women. PLoS ONE.

[21] Murray Stephen A., Morgan Judith L., Kane Coleen, Sharma Yashoda, Heffner Caleb S., Lake Jeffrey, Donahue Leah Rae (2010). Mouse Gestation Length Is Genetically Determined. PLoS ONE.

[22] McCarthy, R. *et al*. Mouse models of preterm birth: Suggested assessment and reporting guidelines. *Biology of Reproduction*, 10.1093/biolre/ioy109 (2018). 10.1093/biolre/ioy109 PMC6297318 29733339

[23] Coan Philip M., Ferguson-Smith Anne C., Burton Graham J. (2004). Developmental Dynamics of the Definitive Mouse Placenta Assessed by Stereology1. Biology of Reproduction.

[24] Rossant Janet, Cross James C. (2001). Placental development: Lessons from mouse mutants. Nature Reviews Genetics.

[25] Scholzen, T. & Gerdes, J. The Ki-67 protein: From the known and the unknown. *Journal of Cellular Physiology*, https://doi.org/10.1002/(SICI)1097-4652(200003)182:3<311::AID-JCP1>3.0.CO;2-9 (2000). 10.1002/(SICI)1097-4652(200003)182:3<311::AID-JCP1>3.0.CO;2-9 10653597

[26] Poespoprodjo Jeanne Rini, Fobia Wendy, Kenangalem Enny, Lampah Daniel A., Warikar Noah, Seal Andrew, McGready Rose, Sugiarto Paulus, Tjitra Emiliana, Anstey Nicholas M., Price Ric N. (2008). Adverse Pregnancy Outcomes in an Area Where Multidrug‐ResistantPlasmodium vivaxandPlasmodium falciparumInfections Are Endemic. Clinical Infectious Diseases.

[27] Rodrigues-Duarte Lurdes, de Moraes Luciana, Barboza Renato, Marinho Claudio RF, Franke-Fayard Blandine, Janse Chris J, Penha-Gonçalves Carlos (2012). Distinct placental malaria pathology caused by different Plasmodium berghei lines that fail to induce cerebral malaria in the C57BL/6 mouse. Malaria Journal.

[28] McDonald Chloe R., Cahill Lindsay S., Gamble Joel L., Elphinstone Robyn, Gazdzinski Lisa M., Zhong Kathleen J. Y., Philson Adrienne C., Madanitsa Mwayiwawo, Kalilani-Phiri Linda, Mwapasa Victor, ter Kuile Feiko O., Sled John G., Conroy Andrea L., Kain Kevin C. (2018). Malaria in pregnancy alters l-arginine bioavailability and placental vascular development. Science Translational Medicine.

[29] Conroy Andrea L., Silver Karlee L., Zhong Kathleen, Rennie Monique, Ward Peter, Sarma J. Vidya, Molyneux Malcolm E., Sled John, Fletcher Joseph F., Rogerson Stephen, Kain Kevin C. (2013). Complement Activation and the Resulting Placental Vascular Insufficiency Drives Fetal Growth Restriction Associated with Placental Malaria. Cell Host & Microbe.

[30] Wilson, M. E. & Ford, S. P. Comparative aspects of placental efficiency. *Reprod Suppl* (2001). 11980192

[31] Takem Ebako Ndip, D'Alessandro Umberto (2013). MALARIA IN PREGNANCY. Mediterranean Journal of Hematology and Infectious Diseases.

[32] Ayres Pereira Marina, Mandel Clausen Thomas, Pehrson Caroline, Mao Yang, Resende Mafalda, Daugaard Mads, Riis Kristensen Anders, Spliid Charlotte, Mathiesen Line, E. Knudsen Lisbeth, Damm Peter, G. Theander Thor, R. Hansson Stefan, A. Nielsen Morten, Salanti Ali (2016). Placental Sequestration of Plasmodium falciparum Malaria Parasites Is Mediated by the Interaction Between VAR2CSA and Chondroitin Sulfate A on Syndecan-1. PLOS Pathogens.

[33] Peltier Morgan R (2003). Reproductive Biology and Endocrinology.

[34] Evseenko D. A., Paxton J. W., Keelan J. A. (2007). Independent Regulation of Apical and Basolateral Drug Transporter Expression and Function in Placental Trophoblasts by Cytokines, Steroids, and Growth Factors. Drug Metabolism and Disposition.

[35] Ogawa Masaki, Hirano Hideto, Tsubaki Hiromitsu, Kodama Hideya, Tanaka Toshinobu (1998). The role of cytokines in cervical ripening: Correlations between the concentrations of cytokines and hyaluronic acid in cervical mucus and the induction of hyaluronic acid production by inflammatory cytokines by human cervical fibroblasts. American Journal of Obstetrics and Gynecology.

[36] Aye Irving L.M.H., Waddell Brendan J., Mark Peter J., Keelan Jeffrey A. (2010). Placental ABCA1 and ABCG1 transporters efflux cholesterol and protect trophoblasts from oxysterol induced toxicity. Biochimica et Biophysica Acta (BBA) - Molecular and Cell Biology of Lipids.

[37] Ietta F., Todros T., Ticconi C., Piccoli E., Zicari A., Piccione E., Paulesu L. (2002). Macrophage Migration Inhibitory Factor in Human Pregnancy and Labor. American Journal of Reproductive Immunology.

[38] Yang, Y. *et al*. Profiling of differentially expressed genes in sheep T lymphocytes response to an artificial primary Haemonchus contortus infection. *Parasites and Vectors*, 10.1186/s13071-015-0844-z (2015). 10.1186/s13071-015-0844-z PMC4406218 25903558

[39] Dunk CE (2018). P‐Glycoprotein (P‐gp)/ABCB1 plays a functional role in extravillous trophoblast (EVT) invasion and is decreased in the pre‐eclamptic placenta. J. Cell. Mol. Med..

[40] Georgiades P., Ferguson-Smith A.C., Burton G.J. (2002). Comparative Developmental Anatomy of the Murine and Human Definitive Placentae. Placenta.

[41] Silva-Filho João Luiz, Souza Mariana Conceição, Ferreira-DaSilva Claudio Teixeira, Silva Leandro Souza, Costa Maria Fernanda Souza, Padua Tatiana Almeida, Henriques Maria das Graças, Morrot Alexandre, Savino Wilson, Caruso-Neves Celso, Pinheiro Ana Acacia Sá (2013). Angiotensin II Is a New Component Involved in Splenic T Lymphocyte Responses during Plasmodium berghei ANKA Infection. PLoS ONE.

[42] Bloise Enrrico, Lin Wingka, Liu Xiaowei, Simbulan Rhodel, Kolahi Kevin S., Petraglia Felice, Maltepe Emin, Donjacour Annemarie, Rinaudo Paolo (2012). Impaired Placental Nutrient Transport in Mice Generated byin VitroFertilization. Endocrinology.

[43] Livak KJ, Schmittgen TD (2001). Analysis of relative gene expression data using real-time quantitative PCR and the 2(−Delta Delta C(T)) Method. Methods.

[44] TSCHANZ S.A., BURRI P.H., WEIBEL E.R. (2011). A simple tool for stereological assessment of digital images: the STEPanizer. Journal of Microscopy.

[45] MORAES ALAN CN DE, ANDRADE CHERLEY BV, SALATA CAMILA, NASCIMENTO ANA LR, RAMOS ISALIRA P, GOLDENBERG REGINA CS, CARVALHO JORGE J, MACHADO ANA CS (2015). A combination of stereological methods, biochemistry and electron microscopy for the investigation of drug treatment effects in experimental animals. Journal of Microscopy.

[46] Festing, M. F. W. Design and statistical methods in studies using animal models of development. *ILAR J* (2006). 10.1093/ilar.47.1.5 16391426

[47] Coan P. M., Angiolini E., Sandovici I., Burton G. J., Constância M., Fowden A. L. (2008). Adaptations in placental nutrient transfer capacity to meet fetal growth demands depend on placental size in mice. The Journal of Physiology.

[48] Hirai, T., Fukui, Y. & Motojima, K. PPARalpha agonists positively and negatively regulate the expression of several nutrient/drug transporters in mouse small intestine. *Biol*. *Pharm*. *Bull*, JST.JSTAGE/bpb/30.2185 (2007). 10.1248/bpb.30.2185 17978498

[49] Murakami Masaaki, Harada Masaya, Kamimura Daisuke, Ogura Hideki, Okuyama Yuko, Kumai Noriko, Okuyama Azusa, Singh Rajeev, Jiang Jing-Jing, Atsumi Toru, Shiraya Sayaka, Nakatsuji Yuji, Kinoshita Makoto, Kohsaka Hitoshi, Nishida Makoto, Sakoda Saburo, Miyasaka Nobuyuki, Yamaguchi-Takihara Keiko, Hirano Toshio (2013). Disease-Association Analysis of an Inflammation-Related Feedback Loop. Cell Reports.

[50] Merrell M. D., Nyagode B. A., Clarke J. D., Cherrington N. J., Morgan E. T. (2013). Selective and Cytokine-Dependent Regulation of Hepatic Transporters and Bile Acid Homeostasis during Infectious Colitis in Mice. Drug Metabolism and Disposition.

[51] Zammit Nathan W., Tan Bernice M., Walters Stacey N., Liuwantara David, Villanueva Jeanette E., Malle Elisabeth K., Grey Shane T. (2013). Low-Dose Rapamycin Unmasks the Protective Potential of Targeting Intragraft NF-κB for Islet Transplants. Cell Transplantation.

[52] Gong Zu-Kang, Wang Shuang-Jie, Huang Yong-Qi, Zhao Rui-Qiang, Zhu Qi-Fang, Lin Wen-Zhen (2014). Identification and validation of suitable reference genes for RT-qPCR analysis in mouse testis development. Molecular Genetics and Genomics.

[53] Coughlin, B. *et al*. Connecting the innate and adaptive immune responses in mouse choroidal neovascularization via the anaphylatoxin C5a and γδT-cells. *Sci*. *Rep*, 10.1038/srep23794 (2016). 10.1038/srep23794 PMC4814842 27029558

