# Supplementary material for: Dysregulation of placental ABC transporters in a murine model of malaria-induced preterm labor
Source: Sci Rep. 2019 Aug 7;9:11488. doi: 10.1038/s41598-019-47865-3 (PMC6685947; doi:10.1038/s41598-019-47865-3)
Supplement: Supplementary file 1 — Sypplementary table 1 [file 41598_2019_47865_MOESM1_ESM.docx]

**Dysregulation of placental ABC transporters in a murine model of malaria-induced preterm labor**

Fontes KN^1^, Reginatto MW^1^, Silva NL^1^, Andrade CBV^1^, Bloise FF^1^, Monteiro VRS^1^, Silva-Filho JL^2,9^, Imperio GE^1,4^, Pimentel-Coelho PM^3^, Pinheiro AAS^2^, Matthews SG^4,5,6,7^, Bloise E^8^, Ortiga-Carvalho TM^1*^.

Laboratory of Translational Endocrinology^1^; Laboratory of Immunology and Biochemistry of Parasitic Diseases^2^; Laboratory of Cellular and Molecular Neurobiology^3^; Institute of Biophysics Carlos Chagas Filho, Federal University of Rio de Janeiro, Rio de Janeiro, Brazil. Department of Physiology^4^; Department of Obstetrics & Gynaecology^5^; Department of Medicine^6^, Faculty of Medicine, University of Toronto, Toronto, Canada. Lunenfeld-Tanenbaum Research Institute^7^, Mount Sinai Hospital, Toronto, Canada. Department of Morphology^8^, Federal University of Minas Gerais, Belo Horizonte, Brazil. Laboratory of Tropical Diseases^9^, Department of Genetics, Evolution, Microbiology and Immunology, Institute of Biology, State University of Campinas, Campinas, Brazil.

**Supplementary Table 1: Pilot experiments**

| **Dose (infected erythrocytes)** | **Pregnant**  **mice (n)** | **% of vaginally born fetuses** |
| --- | --- | --- |
| **1x10^5^** | 2 | 0 (0/9) |
| **5x10^5^** | 2 | 12.5 (1/8) |
| **1x10^6^** | 2 | 100 (10/10) |
